# Facilitating future implementation and translation to clinical practice: The Implementation Planning Assessment Tool for clinical trials

**DOI:** 10.1017/cts.2022.467

**Published:** 2022-10-06

**Authors:** Christine P. Kowalski, Linda M. Kawentel, Tassos C. Kyriakides, Lori Davis, Nicholas W. Bowersox, Amy M. Kilbourne, Grant D. Huang, Andrea L. Nevedal

**Affiliations:** 1 Center for Clinical Management Research, VA Ann Arbor Healthcare System, Ann Arbor, MI, USA; 2 Center for Evaluation and Implementation Resources, VA Ann Arbor Healthcare System, Ann Arbor, MI, USA; 3 Cooperative Studies Program Coordinating Center, VA CT Healthcare System, West Haven, CT, USA; 4 Tuscaloosa VA Medical Center Research Service, Tuscaloosa, AL, USA; 5 Department of Psychiatry and Behavioral Neurobiology, University of Alabama Heersink School of Medicine, Tuscaloosa, AL, USA; 6 Department of Learning Health Sciences, University of Michigan, Ann Arbor, MI, USA; 7 Office of Research and Development, Veterans Health Administration, Washington, DC, USA

**Keywords:** Implementation science, clinical trials, veterans, evidence-based innovations translational science

## Abstract

Implementation assessment plans are crucial for clinical trials to achieve their full potential. Without a proactive plan to implement trial results, it can take decades for one-fifth of effective interventions to be adopted into routine care settings. The Veterans Health Administration Office of Research and Development is undergoing a systematic transformation to embed implementation planning in research protocols through the Cooperative Studies Program, its flagship clinical research program. This manuscript has two objectives: 1) to introduce an Implementation Planning Assessment (IPA) Tool that any clinical trialist may use to facilitate post-trial implementation of interventions found to be effective and 2) to provide a case study demonstrating the IPA Tool’s use. The IPA Tool encourages study designers to initially consider rigorous data collection to maximize acceptability of the intervention by end-users. It also helps identify and prepare potential interested parties at local and national leadership levels to ensure, upon trial completion, interventions can be integrated into programs, technologies, and policies in a sustainable way. The IPA Tool can alleviate some of the overwhelming nature of implementation science by providing a practical guide based on implementation science principles for researchers desiring to scale up and spread effective, clinical trial-tested interventions to benefit patients.

## Introduction

The majority of evidence-based treatments do not become adopted in routine clinical care [1,2], and when they do, they take much too long to become integrated into routine clinical practice. In fact, research has suggested that it takes 17 years to turn only about 14 percent of original research into routine patient care [3,4], and the majority of implementation failures are often rooted in context [5,6]. Key reasons for why clinical trial outcomes fail to translate into practice include lack of relevance to patient quality of life and treatment preferences, provider lack of time, tools, or training, cost of implementation, lack of a purveyor, and healthcare organizational barriers such as lack of incentives, processes, or technologies to facilitate treatment use by frontline providers over time [7]. Such factors often are not accounted for in the design of clinical trials. Adoption of research results into clinical practice and guidelines is a vital component for a learning healthcare system, including the VA.

Implementation science, “the study of methods to promote the adoption and integration of evidence-based practices, interventions, and policies into routine healthcare and public health settings to improve the impact on population health” [8], can help solve this gap. Implementation science utilizes strategies that facilitate provider adoption of interventions that are proven effective in the post-trial period, especially when faced with resource constraints. Implementation strategies are highly specified, theory-based tools, or methods used by organizations or providers to facilitate adoption of an effective treatment.

Despite the value of implementation science, most clinical trials in the United States are not designed to take implementation planning into account. Likewise, to date VA’s clinical trial evaluation approach has not included translational steps to support comprehensive implementation planning and assessment of effective treatments once the trial is completed. Without a specified process for existing providers and sites to ensure trial results are adopted in routine practice by multi-level interested parties once the study is completed, scale up and spread is very difficult. Implementation planning assessments (IPAs) address this lack of uptake by utilizing real-world data to understand the uptake, use, and effectiveness of the intervention, thereby ensuring that evidence-based interventions are set up for successful implementation in clinical practice.

The Veterans Health Administration (VHA) Cooperative Studies Program (CSP), with its long history and tradition of comparative effectiveness and process-driven thinking, offers an ideal opportunity to develop and deploy an implementation plan. For over 90 years, the VHA Office of Research and Development (ORD) has supported groundbreaking multisite clinical trials, most notably through CSP [9]. Historically, CSP has developed and deployed multisite clinical trials within VHA and the nation by using a model that involves multi-disciplinary teams of VHA investigators and national program office leaders to support buy-in and application of trial results within VHA. CSP has also used a standardized robust process evaluation [10] to ensure fidelity to trial protocol, design, and internal validity which includes commonly used quality assurance processes and also meeting registration requirements under the International Organization for Standardization 9001 criteria.

In 2019, VHA ORD instituted a requirement that all CSP clinical trials include an Implementation Plan as a condition of funding to ensure trial results are adopted in clinical practice in VHA and beyond. This requirement stemmed from the Chief Research and Development Officer’s strategic priority to increase the substantial real-world impact of research and to support the use of the ORD VA Research Lifecycle Framework [11]. This Framework maps how clinical trials can incorporate planning and data collection to ensure interventions, if proven effective, are ready to be used by frontline providers in routine care settings. Accordingly, CSP adopted requirements that all new trials include an Implementation Plan, which outlined a process for preparing treatment interventions for their implementation in the aftermath of an effective trial. The Implementation Plan prompts more in-depth data collection on usability and acceptance of the intervention during the trial, while also planning for sustainment by identifying opportunities to embed the effective treatment into routine care, programs, policies, or through marketing.

VHA studies that have not had an implementation plan have struggled to use trial results to impact real-world clinical practice. For example, a VA CSP study published in 2018 showed superior efficacy of a rigorous model of supported employment, called Individual Placement and Support (IPS), for unemployed Veterans with a diagnosis of PTSD compared to usual vocational rehabilitation services [12]; however, VHA has yet to broadly disseminate IPS services to the vast PTSD population beyond a handful of medical centers. In retrospect, proactive implementation science tools may have accelerated the pace of real-world service delivery of the most efficacious treatment.

Current national efforts to move trial results to implementation have been hampered by a lack of a detailed process for embedding implementation science methods into clinical trials. Current research, notably from the experience of the Clinical and Translational Science Awards (CTSAs), outlines a foundation that describes the need for national standards of embedding implementation science into clinical research [13,14]. To address the gap between VA CSP trials and recent implementation plan requirements, we developed the CSP IPA Tool, which has become the basis of CSP Implementation Plans.

The objective of this manuscript is to present the new IPA Tool (Table 1), which provides step-by-step guidance that can be used by trialists in healthcare settings to facilitate future implementation and translation to clinical practice of trial results that support the effectiveness/efficacy of the tested treatment or intervention. To further showcase the practical application of the IPA Tool, we provide a real-world case example from a CSP clinical trial to illustrate the types of data and steps required to complete an IPA (Table 2). Findings from use of our IPA Tool will inform how the trial intervention, if proven effective, can be further deployed across the VA system and beyond to reduce the gap between research and real-world practice. Altogether, these activities are to serve as foundational elements for a broader enterprise-wise strategy for VA-funded studies.

**Table 1.** The Implementation Planning Assessment Tool^1^

**Implementation Planning Assessment Tool for the Veterans Health Administration (VHA) Cooperative Studies Program (CSP) and Other Clinical Trials**
Guidance: As a team, review and document responses to the overarching questions. The intention is for this tool to be completed as an iterative process and the teams and individuals can and should refer to the tool at different points in time throughout the trial.
**Phase 1. Planning, Framing, and Aligning Interested Parties: How do CSP trial programs identify and align interested parties?** Planning, framing, and aligning interested parties helps inform the design of the intervention to be implemented (e.g., design-for implementation, user-centered design). Clinical trial programs often include national interested parties upfront on their Executive Committees, such as national program office leads (e.g., Pharmacy Benefits Management, National Pathology & Laboratory Medicine, Patient Care Services, Clinical Services program offices). However, these programs should also consider identifying and collecting input from potential end-users at the regional (e.g., Veterans Integrated Service Network (VISN), Chief Medical officers (CMOs)) and local levels (e.g., facility Chiefs of Staff and Service Line Chiefs), and Veteran or patient users throughout the trial process. Involving interested parties at multiple levels will also help enhance equity and diversity in implementation planning and garner buy-in, at the local clinic (e.g., frontline provider, facility service line) and regional managerial levels (e.g., VISN Director, CMO).
**1. What is the intervention/treatment and what are its core elements that are hypothesized to achieve its desired effect on health?** Intervention: Core Elements: Desired Effect on Health:
**2. What clinical issue or public health problem is the intervention trying to solve?** Clinical Issue: Intervention:
**3. Create a list of the key interested parties involved in the intervention/treatment (please see Table 3 for a list of potential interested parties). Consider including those with high interest in the treatment or intervention, as well as key influencers in its adoption and sustainment in routine practice over time, and those who may provide insight on equity and diversity considerations. For example, clinical and non-clinical staff, local and regional managers, national program office leads, policymakers, and patients with diverse backgrounds (race/ethnicity, gender, LGBTQ+, disability) in trials. The VA Women’s Enhanced Recruitment Process (WERP) is an example of an effort to increase women’s participation in trials.^2^:**
**4. What staff and resources do you have to support preliminary implementation planning work and delivery of the intervention/treatment?** a. For planning of the study including treatment or intervention/treatment delivery. b. For capturing data on the process by which the treatment is being implemented, such as provider delivery and fidelity to the treatment (e.g., checklists of treatment core elements completed and delivered to each patient). For data capture of the treatment use by existing sources for future surveillance (e.g., Electronic Health Record (EHR), diagnostic, lab, treatment codes). For capturing interested party perspectives of the treatment (patients providers, managers, leaders), such as surveys, interview guides, software.,. Consider including Full-Time Equivalent (FTE) for these tasks. c. Consider staff FTE, protected time, training, supplies (audio recorder, headset, qualitative data analysis software), and equity and diversity of facilities or staff implementing the intervention.
**5. What level of facility, regional, and national leadership support is there for the intervention (describe from high to low)?** a. For facility leadership explain the type of support that leadership is providing for the intervention/treatment (e.g., FTE for clinical trial, prioritizing and spreading support for the intervention or treatment trial recruitment, etc.). *Note: This step includes assessing the current leadership support at involved sites (facilities) and using strategies to increase that support as necessary^1^ Qualitative* *interviews, focus groups, or telephone conversations are methods often used to obtain feedback from leadership.* National: National and Local Site Collaboration: Local Site Support:
**6. Have frontline users (clinical and non-clinical staff) provided input on the design and deployment of the intervention?** *Note: Qualitative interviews, focus groups, or conversations are methods often used to obtain feedback from frontline users* ^3^
**7. Which determinants framework^3^ will be used to identify and describe contextual factors that could influence the implementation process and quality of intervention protocol delivery?** a. The framework informs data collection and analysis to identify barriers and facilitators to practice uptake. *Note: Examples of determinants frameworks include Theoretical Domains Framework (TDF), Consolidated Framework for Implementation Research (CFIR) [19], and the* *integrated-Promoting Action on Research Implementation in Health Services (i-PARIHS) [20].*
**8. Who are the multi-level interested parties of the intervention (e.g., those who have an interest in using the treatment or intervention if proven effective, those who may provide insight on equity and diversity considerations of individuals and/or healthcare settings, and/or those who have influence on the policies that would foster sustainment of the treatment in routine practice if proven effective)?** a. Describe how interested parties were identified and how their perspectives will be assessed over the course of the trial at each participating site (e.g., qualitative or quantitative methods). b. Identify the local and regional interested parties involved in the intervention. c. Map out who needs to take different steps (role) and when in the process for the intervention to be successfully implemented. d. Identify Veteran or patient interested parties. Interested Party Input: Site Selection: Post-selection:
**9. How have multi-level interested parties who have an interest in the intervention/treatment provided input? Interested parties should include Veteran or patient perspectives as well. For all interested parties, elicit feedback about equity, diversity, inclusion, barriers, facilitators, and satisfaction with the intervention/treatment.** a. Consider local, regional, national interested parties. *Note: Qualitative interviews, focus groups, or conversations, advisory calls, are methods often used to obtain feedback from key interested parties.* ^3^
**10. Which national program officers and operational partners could facilitate pathways for future spread or implementation?**
**11. Draft an implementation plan and ensure that your implementation plan includes each of the following important components:** a. The rationale for the selection of appropriate implementation science theories or frameworks. b. Methods for quantitative (e.g., intervention uptake based on existing EHR data), qualitative, or mixed methods data collection and analyses. If using mixed methods, how will the methods be integrated? c. Quantitative sampling plan for control and comparison groups and data codes and fields to capture use in the EHR. CSP implementation leads should coordinate with CSP coordinating centers to determine what data is already being collected to avoid duplication. If not applicable, describe the rationale. d. Qualitative sampling plan of patient and clinical interested parties (e.g., purposeful criterion, stratified, snowball strategies). If not applicable, describe the rationale. e. Specification of the types (quantitative or qualitative) or sources of data (e.g., primary or secondary) to be used, data accessibility, aggregate and subgroup analyses, and provisions for ensuring data quality and adherence to the study protocol.
**12. What are the preliminary plans for the interventions’ sustainment, once the trial ends, if the intervention/treatment is found effective (see also Phase 3)?** The plan should take into consideration any administrative or policy changes needed at the national and regional levels (e.g., formularies, labs, EHR fields, national directives, or other services policies), time, tools, and training required by clinicians at the frontline to deliver the intervention and where (e.g., primary care, specialty care clinics, Community-Based Outpatient Clinics, etc.) and Veteran time required (e.g., visits, required lab tests, medications, etc.) a. The plan should also take into consideration how pragmatic the trial is (e.g., factors that could impact use of the intervention in real-world settings such as cost, intervention deliverers, intervention recipients).
**Phase 2. Implementation Process Data Collection: How will the implementation process be studied, measured, and assessed?** The Implementation Process Data Collection Phase involves ascertainment of factors affecting the use of the CSP intervention or treatment at the routine practice level, notably through information on provider and patient perspectives and acceptance, implementation and intervention costs and organizational factors, and where relevant fidelity to the implementation of the intervention or treatment. This phase also involves enacting an implementation assessment plan and should include equity and diversity considerations throughout.
**1. Who is part of your assessment team?** Describe the amount and type of time set-aside for the assessment team, implementation lead, or others (e.g., FTE, protected time, donated).
**2. Have you finalized your assessment plan (#11 in planning) to address the following?** a. The rationale for the selection of appropriate implementation science theories or frameworks. b. Methods for quantitative, qualitative, or mixed methods data collections and analyses. If using mixed methods, how will the methods be integrated? c. Quantitative sampling plan for control and comparison groups for describing the implementation process and intervention uptake and use. If not applicable, describe the rationale. d. Qualitative sampling plan of patient, local-, regional- and national-level leadership, and clinical interested parties, including specific techniques and measures. If not applicable, describe the rationale. e. Specification of the kinds or sources of data to be used, data accessibility, aggregate and subgroup analyses, and provisions for ensuring data quality and adherence to the study protocol.
**3. Which implementation strategies did you select to help attain successful implementation of the intervention?** a. For efficacy trials, define your implementation strategies and their goals. b. For effectiveness trials, is the trial paying for providers to deliver the treatment/ intervention to the patients or relying on existing providers? How do you intend to measure: 1) fidelity to the intervention or treatment, 2) the uptake or use of the treatment (e.g., patient use or “dose” and 3) what will training, and competencies look like for existing providers once the study ends and the intervention is shown to be effective (e.g., for providers to take up the effective intervention, what level of expertise and training is optimal and what will the manual contain on how they deliver the treatment?) 4) identify the costs of training, 5) consider interested party buy-in for training c. Document data about your implementation strategy enactment that could help future sites during scale-up and spread if the intervention is found effective d. To what extent have interested parties provided input into the selection of the implementation strategies? e. Describe the dose of implementation strategies [16]. *Note: Selection of implementation strategies will be based on the key barriers and key facilitators identified during pre-implementation.*
**4. What adaptations or resources are needed for to fit local contexts?** Please note for efficacy trials, adaptations may be less relevant or applicable. a. Describe the *core components* of the clinical intervention or treatment that are believed to be the causal mechanisms of therapeutic change and are responsible for achieving the intervention or treatment’s desired effects (those facets of the intervention that cannot be adapted or changed). b. Explain how the intervention can be adapted without compromising fidelity. c. Consider how adaptations can address or increase equitable implementation of the innovation.
**5. What are the benchmarks of successful implementation?** a. Explain how successful implementation will be measured. b. Describe how the impact of dissemination or implementation will be measured. c. Include patient-perspective benchmarks as well, including health equity, barriers, and satisfaction.
**6. How is the intervention perceived and used by key interested parties?** a. Consider frontline providers/staff, local leadership, and patients. b. Elicit feedback from interested parties about equity and diversity concerns regarding implementation of the intervention or treatment. *Note: This will include conversations and/or qualitative interviews with frontline users (overlap with and/or modified from pre-implementation questions.*
**7. What is the plan for assessment of the uptake and fidelity in delivery of the treatment or intervention?** a. Describe how the uptake or use of the intervention or treatment by the patients is measured (ideally, using EHR data) b. Describe who is responsible for assessing fidelity of the treatment or intervention delivery by the provider. How will fidelity be assessed among existing providers (i.e., those not funded by the study)
**8. What preliminary insight can key interested parties (patient/Veteran as well as clinical interested parties, including local-, regional- and national-level leadership) provide about barriers to sustainment (to inform phase 3)?**
**Phase 3. Planning for Sustainment for Effective Trials: If the intervention is found to be effective, how will the intervention be spread to new sites and sustained by interested parties?** If the intervention is found to be effective, it is important for there to be sustainment planning activities in place so that the intervention is maintained and continuously provides benefits to the healthcare system. Sustainment planning will help evaluators/researchers understand how the intervention will be used in routine care once the study has ended and if there are important equity and diversity considerations needed for sustainment. Results from Phase 1 and 2 will inform clinicians and healthcare sites in understanding how to deliver the intervention protocol more effectively, make appropriate adaptations, and sustain the intervention over time. This can be written as a “toolkit” or “implementation playbook” that outlines next steps for national policy and organization changed and recommendations for provider training and delivery. This “toolkit,” built during the course of the trial, can then be deployable to other (new) sites if the intervention is found effective.
**1. How can results from Phases 1 and 2 be used to develop an implementation strategy, process, or “playbook” by which interventions will be adopted and sustained in routine practice by existing providers?** a. Describe the extent to which the trial was pragmatic and its implications for sustainment (i.e., if it was not pragmatic, how will might this impact sustainment and scale-up of the intervention or treatment? b. Describe the training required among existing and new providers/staff to deliver the treatment or intervention c. Describe the clinical processes required to maintain the treatment or intervention in routine practice and what additional administrative changes might be needed (e.g., addition of treatment into VA national formulary, lab tests, additional clinic time or procedures required to deliver treatment), where the intervention will take place (e.g., primary care, specialty, etc.), and how sustainable the intervention is (i.e., consider resources and staffing once the trial ends). d. Determine which measures should be used to monitor use of the intervention or treatment in routine practice (e.g., from existing EHR data, addition of variables to the formulary/EHR, etc. e. Identify how sustainment of the intervention can be tracked over time (e.g., dashboards, surveys). f. Consider how qualitative data from leadership or interested parties can be used to understand the factors that may help or hinder future “real-world” spread, implementation, or sustainment and how there may be unique equity and diversity considerations during these different phases g. Describe if certain implementation strategies are more likely to sustain this intervention over time and have sustained effects over time. h. Consider how results will inform future interested parties’ acceptance of the intervention. i. Collect information on cost, including opportunity costs, and burden from different interested party perspectives, patient engagement, satisfaction, barriers, and health equity. *Note: Qualitative interviews or surveys with intervention-interested parties during phase 1 and phase 2 could be used to understand potential sustainment of the* *intervention3*
**Phases 1–3. Planning for Dissemination: How will intervention and implementation information and trial results be shared with others to increase adoption of the intervention?** Throughout all 3 phases of implementation planning, teams should consider the types of information that should be disseminated, to whom information should be disseminated, and how information should be tailored to address equity and diversity considerations of different individuals and healthcare settings. Dissemination is important for increasing awareness of the intervention, offering opportunities for bidirectional communication, and accelerating the buy-in/adoption/uptake of the intervention by providers, patients, and/or healthcare systems.
**1. What information should be disseminated during Phase 1 “Planning, Framing, and Aligning Interested parties” (e.g., increase intervention awareness and buy-in among interested parties)?** a. Identify to whom information should be disseminated by creating a visual display or mapping of interested parties b. Consider dissemination as an opportunity for bidirectional communication to and from interested parties to inform initial planning
**2. What information should be disseminated during Phase 2 “Implementation Process and Data Collection” (e.g., Share implementation plans and support tools with key interested parties)?** a. Identify to whom information should be disseminated by creating a visual display or mapping of interested parties b. Consider dissemination as an opportunity for bidirectional communication to and from interested parties to inform implementation
**3. What information should be disseminated during Phase 3 “Planning for Sustainment for Effective Trials” (e.g., maintain priority and awareness of the intervention among key interested parties)?** a. Identify to whom information should be disseminated by creating a visual display or mapping of interested parties b. Consider dissemination as an opportunity for bidirectional communication to and from interested parties to inform sustainment
**4. Throughout all three phases, what is the plan for how and when intervention information and trial results will be disseminated, tailored, and communicated to interested parties and potential non-VA adopters? The following considerations may differ for each of the three phases:** a. Identify various “passive” publication opportunities (e.g., peer-reviewed journals, other publications) b. Identify more “active” strategies for disseminating information and results, which have been found to be more effective in reaching key interested parties (e.g., briefings to VA local, regional and national leaders-CMO national calls, VISN meetings, program office meetings, news or social media outlets, workshops, meetings) c. Determine if there are local or national opportunities to present trial results (e.g., professional conferences, VHA Cyber-seminars) d. Consider how intervention materials and results can be disseminated to diverse interested parties (e.g., clinical and non-clinical audiences, patient groups, Veteran and Family Advisory Councils). e. Develop other products that could be used to disseminate trial results (e.g., websites, toolkits, playbooks) *Note: Consider developing an interested parties map to identify and engage relevant interested parties in future implementation processes. B-E will likely offer more* *opportunities for bidirectional communication*
**5. What are the plans for using the intervention in future implementation research?** *Note: Consider funding from a VA QUERI center, VA Office of Research and Development/Health Services Research & Development (HSR&D) research study, VA national* *program office policy (use the Evidence Act), VA Diffusion of Excellence, National Institutes of Health-funded research, or foundation grants.*

1 While the tool was designed for use in both efficacy and effectiveness trials, effectiveness trials will have broader and more involved implementation methodology since they focus on answering the question: do the intervention benefits hold true in real-world clinical settings? In contrast, the tool will be more limited in efficacy trials since they focus on the answering the question: does the intervention work in a highly controlled standardized setting? Therefore, throughout the tool, we have indicated sections that may not be useful during efficacy trials.

2 See Frayne SM, Pomernacki A, Schnurr PP. *Women’s Enhanced Recruitment Process (WERP): Experience with Enhanced Recruitment of Women Veterans to a CSP Trial.* Invited national VA HSR&D CyberSeminar, presented November 15, 2018. https://www.hsrd.research.va.gov/for_researchers/cyber_seminars/archives/video_archive.cfm?SessionID=3565

3 Denotes overlap between Planning, Framing, and Aligning Interested Parties Phase and Implementation Process Data Collection phases.

**Table 2.** Case study example using the Implementation Planning Assessment Tool^1^

**Implementation Planning Assessment Tool for the Veterans Health Administration (VHA) Cooperative Studies Program (CSP) and other clinical trials**
Guidance: As a team, review and document responses to the overarching questions. The intention is for this tool to be completed as an iterative process and the teams and individuals can and should refer to the tool at different points in time throughout the trial.
**Phase 1. Planning, Framing, and Aligning Interested Parties: How do CSP trial programs identify and align interested parties?** Planning, framing, and aligning interested parties help inform the design of the intervention to be implemented (e.g., design-for implementation, user-centered design). Clinical trial programs often include national interested parties upfront on their Executive Committees, such as national program office leads (e.g., Pharmacy Benefits Management, National Pathology & Laboratory Medicine, Patient Care Services, Clinical Services program offices). However, these programs should also consider identifying and collecting input from potential end-users at the regional (e.g., Veterans Integrated Service Network (VISN), Chief Medical officers (CMOs)) and local levels (e.g., facility Chiefs of Staff and Service Line Chiefs), and Veteran or patient users throughout the trial process. Involving interested parties at multiple levels will also help enhance equity and diversity in implementation planning and garner buy-in, at the local clinic (e.g., frontline provider, facility service line) and regional managerial levels (e.g., VISN Director, CMO).
**1. What is the intervention/treatment and what are its core elements that are hypothesized to achieve its desired effect on health?** Intervention: *Individual Placement and Support (IPS) is the evidence-based model of supported employment and is highly effective in helping Veteran patients* *who are diagnosed with post-traumatic stress disorder (PTSD) obtain and sustain employment in competitive jobs.* Core Elements: *An IPS Specialist provides personalized employment services that follow these core elements: (a) zero exclusion for eligibility, (b) rapid job search;* *(c) systematic job development in a diversity of jobs; (d) competitive employment rather than set-aside or transient jobs; (e) client choice for well-matched and meaningful jobs; (f) integration of IPS within the clinical treatment team ensuring shared decision making between providers and Veteran clients; (g) personalized benefits counseling; and (h) individualized support during follow-up for as long as needed even after a competitive job is obtained.* Desired Effect on Health: *To acquire meaningful sustained competitive employment in a well-matched job to help a person with a disability overcome personal,* *professional, and interpersonal difficulties. This is particularly important for someone living with PTSD, since real-world experiences through competitive work helps therapeutically break through the patient’s behavioral isolation/avoidance, cognitive distortions, and emotional reactivity. Employment is often the “hook” to motivate the patient to engage in treatment programs to better their chances of recovery and further success.*
**2. What clinical issue or public health problem is the intervention trying to solve?** Clinical Issue: *Unemployment or occupational difficulty that have broad negative impacts on quality of life, physical and mental health outcomes (including* *increasing the risk of suicide and/or addictions), and societal economic burden.* Intervention: *The IPS intervention aims to positively impact the likelihood of obtaining and maintaining employment, which is a clinically relevant functional* *outcome for a disabling condition such as PTSD. VA Cooperative Study #589 Veterans Individual Placement and Support Toward Achieving Recovery (VIP-STAR) targeted steady competitive employment for Veteran patients, as its primary outcome. Rather than selecting a PTSD symptom outcome, the primary outcome was steady employment, which is a functional outcome that is more relevant to the quality of life and recovery of the Veteran with PTSD. Functional recovery in work sets forth a trajectory which improves psychological outcomes and reduces negative health outcomes over time.*
**3. Create a list of the key interested parties involved in the intervention/treatment (including those with high interest in the treatment or intervention, as well as key influencers in its adoption and sustainment in routine practice over time, and those who may provide insight on equity and diversity considerations, for example, patients, clinical and non-clinical staff, local and regional managers, national program office leads, policymakers, and recruitment of diverse patients (race/ethnicity, gender, LGBTQ+, disability) in trials, for example, the VA Women’s Enhanced Recruitment Process (WERP)^2^):** • *Unemployed Veterans living with a diagnosis of PTSD who have an interest in gaining competitive employment* • *Local facility PTSD care providers and mental health leadership* • *Community employers and workforce development organizations* • *VA’s Office of Mental Health and Suicide Prevention (OMHSP)* • *National Mental Health Director for Psychosocial Rehabilitation and Recovery Services at VHA10NC5ACTION@va.gov* • *VA National Center for PTSD* • *Northeast Program Evaluation Center (NEPEC) in OMHSP* • *Veterans Integrated Service Network Directors and Facility Directors* • *Veterans Integrated Service Network Chief Mental Health Officers and Vocational Rehabilitation Service Program Managers, Staff, and IPS Specialists* • *Veterans Integrated Service Network Supported Employment Mentor-Trainers* • *VA Medical Facility Local Recovery Coordinators* • *IPS Learning Community (non-VA IPS practice leaders in the field)* *See VHA DIRECTIVE 1163 August 13, 2019, for definitions and roles for these* ***interested parties***.
**4. What staff and resources do you have to support preliminary implementation planning work and delivery of the intervention/treatment?** a. For planning of the study including treatment or intervention/treatment delivery. b. For capturing data on the process by which the treatment is being implemented, such as provider delivery and fidelity to the treatment (e.g., checklists of treatment core elements completed and delivered to each patient), data capture of the treatment use by existing sources for future surveillance (e.g., Electronic Health Record (EHR), diagnostic, lab, treatment codes), surveys, interview guides, software, etc. for capturing interested party perspectives of the treatment (patient, provider, manager, leaders). Consider including Full-Time Equivalent (FTE) for these tasks. c. Consider staff FTE, protected time, supplies, and equity and diversity of facilities or staff implementing the intervention (audio recorder, headset, software, training). *The project did not have existing IPS specialists in place at the local site who could serve a full caseload of Veterans with a diagnosis of PTSD. Thus, CSP #589* *funded one or two IPS specialists at each local study site. The CSP Executive Committee’s IPS experts gave input into the selection of the new IPS specialists and at times, the onboarding of the new IPS specialist(s) was held up by Human Resources due to the IPS candidate not having a specific educational degree in vocational rehabilitation counseling. This advanced degree does not necessarily translate into a person with the training, skill set, or understanding of the IPS practice. Advocacy from the local site investigator and CSP Executive Committee IPS experts were needed to facilitate the onboarding of suitable candidates to fill the IPS specialist positions. In addition, the teams had to work together when there was turn-over in the IPS specialist positions or if an IPS incumbent had performance deficits.* *In addition, training, ongoing technical assistance, and fidelity monitoring for the local IPS Specialists were carried out by CSP-funded IPS Trainers and an IPS* *Fidelity Monitor as part of the initial kickoff visit and subsequent on-site visits at the participating sites. This ensured the fidelity of the IPS model was maintained while any barriers of model implementation at the local facility were addressed. For example, bringing together the clinical and research teams at each participating facility ensured first and foremost the research project kept the Veteran participant’s well-being and welfare at its core; no issue went unnoticed irrespective of whether it came from the research team or the clinical team. This in turn, ensured the data processes followed in the research protocol were complete and in sync with clinically collected information, since both teams were aware of the Veteran’s participation in the study and whether they were assigned to IPS or the control intervention.* *The control group intervention (Transitional Work Program) was provided by each local facility’s existing vocational rehabilitation services. The study team had to* *work with some sites to ensure that the control group honored the time-limited nature of transitional work assignments (i.e., the set-aside job needed to be kept within a national standard of approximately 90-day length rather than a protracted 6 months or more that some sites had evolved into over many years prior to the study implementation).* *The CSP study also funded one FTE Clinical Research Coordinator at each site to manage regulatory processes, informed consent, baseline and follow-up* *assessments, data collection, data submission to CSP, and data queries. Each site had a designated local site investigator (not funded by CSP) who was a provider embedded in either the PTSD team or the vocational rehabilitation team.*
**5. What level of facility, regional, and national leadership support is there for the intervention (describe from high to low)?** a. For facility leadership explain the type of support that leadership is providing for the intervention/treatment (e.g., FTE for clinical trial, prioritizing and spreading support for the intervention or treatment trial recruitment, etc.). *Note: This step includes assessing the current leadership support at involved sites (facilities) and using strategies to increase that support as necessary^3^* *Qualitative interviews, focus groups, or telephone conversations are methods often used to obtain feedback from leadership.* ^3^
*National: National leadership provided support in the planning phase. VA CSP established a planning committee that included some national interested parties* *during the design phase. After the study was approved for funding, the VA CSP #589 Executive Committee was formed and included representatives from NEPEC, local study sites, and other IPS experts in VA and non-VA settings. A national consultant was funded by Quality Enhancement Research Initiative (QUERI) to conduct qualitative interviews with the IPS Specialists and local site interested parties during the later stages of the project.* *National and Local Site Collaboration: The CSP project also funded an IPS expert who conducted semi-annual IPS fidelity monitoring visits at the local sites.* *His work at the local site level focused on discussions with the study teams and others at the facility to ensure the IPS intervention was successfully launched and sustained. A fidelity monitoring visit ended with a debrief session with the facility Director, front office Quad members, Service Line Directors, and vocational rehabilitation program managers. These debrief sessions with the facility leadership often included presentations of actual case examples of veteran participants and their lived experiences of prior unemployment and the success of IPS in restoring their treatment goals. Issues were also brought back to the Study Executive Committee for discussion and action as necessary.* *Local Site Support: The local facility leadership provided 1) the protected time for the local site investigators who were responsible for the protocol-related care* *and coordination of the research participants, 2) the PTSD treatment team providers who were responsible for the clinical treatment of the participants, 3) the vocational rehabilitation specialists who provided the control intervention, and 4) authority to work through barriers of study-related processes and IPS implementation when needed (e.g., human resources, information technology, research services, government car motor pool, and space).*
**6. Have frontline users (clinical and non-clinical staff) provided input on the design and deployment of the intervention?** *Note: Qualitative interviews, focus groups, or conversations are methods often used to obtain feedback from frontline users* ^3^ *Clinical and non-clinical input was provided in the planning stages of the study. The CSP Planning Committee was a diverse group of clinicians, methodologists,* *and subject matter experts. Focus groups and interviews did not take place during the planning process; however, these took place in the later stages of the study. Frontline clinical providers (users) were members of the CSP #589 Executive Committee and met quarterly to discuss implementation and enrollment issues. Annually, the local site investigators and IPS specialists gathered in-person to go over the study status and discuss IPS implementation. To allow for in-depth discussions at the annual meetings, breakout meetings were held for investigators and study coordinators to discuss study protocol issues and for IPS specialists to discuss implementation practices).* *More feedback from local site frontline users should have been solicited outside of the IPS* *Fidelity Monitoring visits so that users could have felt more open to share concerns and express difficulties.*
**7. Which determinants framework^3^ will be used to identify and describe contextual factors that could influence the implementation process and quality of intervention protocol delivery?** a. The framework informs data collection and analysis to identify barriers and facilitators to practice uptake. *Note: Examples of determinants frameworks include Theoretical Domains Framework (TDF), Consolidated Framework for Implementation Research (CFIR) [19],* *and the integrated-Promoting Action on Research Implementation in Health Services (i-PARIHS) [20].* *None of the above.* *As a framework, a published structured IPS Fidelity Scale was used to evaluate the IPS implementation, quality, and facilitators/barriers to implementation.* *Fidelity monitoring visits were conducted semi-annually and involved in-depth interviews with leadership, staff, treatment providers, Veterans, and employers, in addition to chart reviews of IPS recipients and observations of team meetings and IPS community interactions with Veterans and employers.*
**8. Who are the multi-level interested parties of the intervention (e.g., those who have an interest in using the treatment or intervention if proven effective, those who may provide insight on equity and diversity considerations of individuals and/or healthcare settings, and/or those who have influence on the policies that would foster sustainment of the treatment in routine practice if proven effective)?** a. Describe how interested parties were identified and how their perspectives will be assessed over the course of the trial at each participating site (e.g., qualitative or quantitative methods). b. Identify the local and regional interested parties involved in the intervention. c. Map out who needs to take different steps (role) and when in the process for the intervention to be successfully implemented. d. Identify Veteran or patient interested parties. *Interested Party Input: The National Mental Health Director for Psychosocial Rehabilitation and Recovery Services (at this time called Director for Compensated* *Work Therapy) and a representative from the Northeast Program Evaluation Center were interested parties involved in the planning for the intervention. The leadership at the National Center for PTSD was kept informed as to the planning of the study and gave informal input. In hindsight, the project would have benefited from more formal input from the VHA’s Office of Mental Health and Suicide Prevention (OMHSP) regarding sustained post-study implementation of IPS.* *Site Selection: An email was sent from CSPCC to the research offices and facility directors at all VA Medical Centers announcing the study and asking for* *interested medical centers to fill out a survey. Based on the results of this survey, size and demographics of the PTSD population, record of success of prior IPS implementation for other populations, track record of research success, presence of a CSP NODE, and local site investigators’ qualifications, 12 sites were identified to serve as the CSP sites.* *Post-selection: Facility leadership, the PTSD care team, and the research team worked together at each participating VA Medical Center to share and discuss the* *implementation of the study and the plan of care for Veterans enrolled in the study.*
**9. How have multi-level interested parties who have an interest in the intervention/treatment provided input? Interested parties should include Veteran or patient perspectives as well. For all interested parties, elicit feedback about equity, diversity, inclusion, barriers, facilitators, and satisfaction with the intervention/treatment.** a. Consider local, regional, national interested parties. *Note: Qualitative interviews, focus groups, or conversations, advisory calls, are methods often used to obtain feedback from key interested parties.* ^3^ *The proposal was reviewed by the Human Rights Committee which has Veteran representation. This was the only instance where Veteran input was provided.* *The investigators had prior experience with IPS implementation and/or with PTSD populations. Qualitative interviews with consumer-interested parties were conducted to evaluate the barriers, and facilitators of IPS implementation were collected over the course of the study (Pogoda QUERI project). At the end of the study, participants completed a satisfaction survey.*
**10. Which national program officers and operational partners could facilitate pathways for future spread or implementation?** • *VA’s Office of Mental Health and Suicide Prevention (OMHSP)* • *VA Psychosocial Rehabilitation and Recovery Services in OMHSP* • *Northeast Program Evaluation Center (NEPEC) in OMHSP* • *VA National Center for PTSD* • *Veterans Integrated Service Network Directors and Facility Directors* • *Veterans Integrated Service Network Chief Mental Health Officers* • *VA QUERI and/or VA Diffusion of Excellence* • *VA Office of the Secretary*
**11. Has your implementation plan included each of the following important components?** a. The rationale for the selection of appropriate implementation science theories or frameworks. ***Yes*** b. Methods for quantitative (e.g., intervention uptake based on existing EHR data), qualitative, or mixed methods data collection and analyses. If using mixed methods, how will the methods be integrated? ***Yes*** c. Quantitative sampling plan for control and comparison groups and data codes and fields to capture use in the EHR. CSP implementation leads should coordinate with CSP coordinating centers to determine what data is already being collected to avoid duplication. If not applicable, describe the rationale. ***N/A; this study preceded the CSP Implementation initiative. Data required for the fidelity monitoring were collected as part of the protocol.*** d. Qualitative sampling plan of patient and clinical **interested parties** (e.g., purposeful criterion, stratified, snowball strategies). If not applicable, describe the rationale. ***No*** e. Specification of the types (quantitative or qualitative) or sources of data (e.g., primary or secondary) to be used, data accessibility, aggregate and subgroup analyses, and provisions for ensuring data quality and adherence to the study protocol. ***This was described in the proposal; monitoring of data and adherence to protocol was carried out through the CSP processes as specified by the study protocol.***
**12. What are the preliminary plans for the interventions’ sustainment, once the trial ends, if the intervention/treatment is found effective (see also Phase 3)?** The plan should take into consideration any administrative or policy changes needed at the national and regional levels (e.g., formularies, labs, EHR fields, national directives, or other services policies), time, tools, and training required by clinicians at the frontline to deliver the intervention and where (e.g., primary care, specialty care clinics, Community-Based Outpatient Clinics, etc.) and Veteran time required (e.g., visits, required lab tests, medications, etc.) a. The plan should also take into consideration how pragmatic the trial is (e.g., factors that could impact use of the intervention in real-world settings such as cost, intervention deliverers, intervention recipients). *No such plans were defined a priori. At semi-annual fidelity debrief sessions, significant efforts were made to present the case for effectiveness of IPS to the sites’* *leadership and encourage the sites to adopt the IPS model for their PTSD population after the study ended. Resource management priorities were often raised as a core challenge by the facility leadership. The leadership valued the IPS model, but resource constraints made it impossible to hire the IPS specialist post-study.* *The plan did not take into consideration that a major policy change would be needed at the national level (i.e., VHA Directive language and IPS service provision* *for a new population of Veterans served). Nor did it accurately gauge the tenacity of the treatment-as-usual vocational services that possibly will require reallocation of resources and/or re-training of existing staff in the transition from non-evidence-based to evidence-based service delivery.*
**Phase 2. Implementation Process Data Collection: How will the implementation process be studied, measured, and assessed?** The Implementation Process Data Collection Phase involves ascertainment of factors affecting the use of the CSP intervention or treatment at the routine practice level, notably through information on provider and patient perspectives and acceptance, implementation and intervention costs and organizational factors, and where relevant fidelity to the implementation of the intervention or treatment. This phase also involves enacting an implementation assessment plan and should include equity and diversity considerations throughout.
**1. Who is part of your assessment team? Describe the amount and type of time set-aside for the assessment team, implementation lead, or others (e.g., FTE, protected time, donated).** *The uptake of the intervention was done in an organic way, via further discussions with the participating site investigators. There was significant activity by the* *Study Chair through discussions/communication with other interested parties that aimed at implementation of the intervention, given its effectiveness and impact on outcomes. Most of this work for post-study implementation was done under the study leadership (i.e., CSP Coordinating Center (CC) and Executive Committee) on donated effort.*
**2. Have you finalized your assessment plan (#11 in planning) to address the following?** a. The rationale for the selection of appropriate implementation science theories or frameworks. b. Methods for quantitative, qualitative, or mixed methods data collections and analyses. If using mixed methods, how will the methods be integrated?. c. Quantitative sampling plan for control and comparison groups for describing the implementation process and intervention uptake and use. If not applicable, describe the rationale. d. Qualitative sampling plan of patient, local-, regional- and national-level leadership, and clinical interested parties, including specific techniques and measures. If not applicable, describe the rationale. e. Specification of the kinds or sources of data to be used, data accessibility, aggregate and subgroup analyses, and provisions for ensuring data quality and adherence to the study protocol. *N/A (retrospective use of checklist)*
**3. Which implementation strategies did you select to help attain successful implementation of the intervention?** a. For efficacy trials, define your implementation strategies and their goals. b. For effectiveness trials, is the trial paying for providers to deliver the treatment/ intervention to the patients or relying on existing providers? How do you intend to measure: 1) fidelity to the intervention or treatment, 2) the uptake or use of the treatment (e.g., patient use or “dose” and 3) what will training, and competencies look like for existing providers once the study ends and the intervention is shown to be effective (e.g., for providers to take up the effective intervention, what level of expertise and training is optimal and what will the manual contain on how they deliver the treatment?) 4) identify the costs of training, 5) consider interested party buy-in for training c. Document data about your implementation strategy enactment that could help future sites during scale-up and spread if the intervention is found effective d. To what extent have interested parties provided input into the selection of the implementation strategies? e. Describe the dose of implementation strategies [16]. *Note: Selection of implementation strategies will be based on the key barriers and key facilitators identified during pre-implementation.* *A structured study-wide process to help attain broad implementation (e.g., implementation at all study sites once the clinical trial ends) was not designed prior to* *study launch for this intervention. This deficit in planning for implementation likely negatively impacted the sustainment of IPS at the 12 local sites. A key barrier to post-study implementation was lack of funding for the local site IPS specialists designated to serve the PTSD patient population. Precedent existed for VA to support an enterprise-wide rollout of IPS for Veterans with psychotic disorders (2005). The investigators assumed that a similar funding stream would materialize after the evidence emerged for the PTSD population. Leadership changes at the national level led to an attrition in resources for IPS sustainment which resulted in diminished focus on expanding services and/or ongoing quality monitoring across the board. Had the checklist been used prospectively, cost planning would have occurred that likely would have enabled planning to circumvent this barrier.* *A key facilitator to sites that did later implement IPS (which were not study sites) was a new federal funding opportunity for IPS implementation (pay-for-success* *social impact bond) involving VA and non-federal partners providing financial support and VA medical centers willing to expand IPS service under the new funding stream.*
**4. What adaptations or resources are needed for to fit local contexts?** Please note for efficacy trials, adaptations may be less relevant or applicable. a. Describe the *core components* of the clinical intervention or treatment that are believed to be the causal mechanisms of therapeutic change and are responsible for achieving the intervention or treatment’s desired effects (those facets of the intervention that cannot be adapted or changed). b. Explain how the intervention can be adapted without compromising fidelity. c. Consider how adaptations can address or increase equitable implementation of the innovation *IPS core principles that are believed to be the mechanisms of therapeutic change and that cannot be adapted or changed: (a) zero exclusion for eligibility;* *(b) rapid job search; (c) systematic job development in a diversity of jobs; (d) competitive employment rather than set-aside or transient jobs; (e) client choice for well-matched and meaningful job; (f) integration of IPS within the clinical treatment team ensuring shared decision making between providers and clients; (g) personalized benefits counseling; and (h) individualized support during follow-up for as long as needed even after a competitive job is obtained.* *Adaptations that can be made without compromising fidelity are related to the specifics of interacting with Veterans who are recovering from PTSD and* *integrating IPS in a new treatment team, that is, the PTSD Clinical Treatment teams. These adaptations are based on PTSD treatment team culture and patients’ behavioral symptoms that differ from the traditional IPS integration with mental health teams that care for Veterans with serious mental illness (i.e., psychotic disorders). For example, compared to Veterans with psychotic disorders, patients living with PTSD tend to be more active in their own job development and can find a job, but are often unable to sustain employment which subsequently requires greater collaboration and partnership between the patient, IPS specialist and PTSD treatment provider.*
**5. What are the benchmarks of successful implementation?** a. Explain how successful implementation will be measured. b. Describe how the impact of dissemination or implementation will be measured. c. Include patient-perspective benchmarks as well, including health equity, barriers, and satisfaction *No metrics were drafted in the study protocol to evaluate these parameters. Future implementation projects should include these measures of success: number of* *Veterans referred for and engaged in IPS services per month, number and duration of competitive jobs gained per participant, income earned per participant, patient satisfaction surveys, IPS fidelity scale scores, and job satisfaction scores.*
**6. How is the intervention perceived and used by key interested parties?** a. Consider frontline providers, local leadership, and patients. b. Elicit feedback from interested parties about equity and diversity concerns regarding implementation of the intervention or treatment. *Note: This will include conversations and/or qualitative interviews with frontline users (overlap with and/or modified from pre-implementation questions.* *During on-site visits, the IPS trainers and fidelity monitor would interview frontline providers, staff, and patients to obtain firsthand information about IPS* *implementation, barriers, and facilitators. There were varied levels of integration of IPS within the PTSD treatment team. For example, providers, who were understanding of and embraced the recovery model and principles of IPS, would make early and ongoing referrals. However, there were providers who were initially reluctant to refer Veterans for fear that the Veteran was “not ready" to return to work or the work would exacerbate the symptoms of PTSD. These fears diminished as case examples proved the resiliency of the Veterans and strengths of individual supports provided by the IPS specialists. Some Veterans had misperceptions that returning to work would jeopardize their VA disability benefits, but these Veterans were provided education on the legal statutes that protect benefits while participating in a VA vocational rehabilitation program. The IPS trainer/fidelity monitor would provide timely information and feedback to the local site investigators and IPS specialists, which helped improve and accelerate implementation of the IPS intervention. The discussion with the providers was done in the context of the various study team meetings. The CSP Study Chair (Davis) made individual site visits to launch the study instead of holding an all-hands national start-up meeting, which helped tailor IPS implementation at the site level since she could meet with many more providers and leadership at the site level instead of only the research team at a national meeting.*
**7. What is the plan for assessment of the uptake and fidelity in delivery of the treatment or intervention?** a. Describe how the uptake or use of the intervention or treatment by the patients is measured (ideally, using EHR data) b. Describe who is responsible for assessing fidelity of the treatment or intervention delivery by the provider. How will fidelity be assessed among existing providers (i.e., those not funded by the study) *The only fidelity monitoring of the intervention was done during the study. No others were carried out once the study ended. The IPS trainer/fidelity monitor has* *over 30 years of IPS implementation experience across community mental health and VA settings. Measurement of fidelity of IPS implementation is essential and scores correlate with patient-level employment outcomes, that is, higher fidelity scores yield better employment outcomes.*
**8. What preliminary insight can key interested parties (patient/Veteran as well as clinical interested parties, including local-, regional- and national-level leadership) provide about barriers to sustainment (to inform phase 3)?** *Note: This data is best collected through qualitative interviews with key interested parties and/or a fidelity or satisfaction survey.* *In assessing model sustainability, feedback and insight would be obtained from involved Veteran participants and their healthcare providers. In addition,* *upstream of these interested parties, information from local/regional and national employment services and/or service chiefs would allow for a synthesis of barriers of not only deployment but long-term viability of the IPS model.*
**Phase 3. Planning for Sustainment for Effective Trials: If the intervention is found to be effective, how will the intervention be spread to new sites and sustained by interested parties?** If the intervention is found to be effective, it is important for there to be sustainment planning activities in place so that the intervention is maintained and continuously provides benefits to the healthcare system. Sustainment planning will help evaluators/researchers understand how the intervention will be used in routine care once the study has ended and if there are important equity and diversity considerations needed for sustainment. Results from Phase 1 and 2 will inform clinicians and healthcare sites in understanding how to deliver the intervention protocol more effectively, make appropriate adaptations, and sustain the intervention over time. This can be written as a “toolkit” or “implementation playbook” that outlines next steps for national policy and organization changed and recommendations for provider training and delivery. This “toolkit,” built during the course of the trial, can then be deployable to other (new) sites if the intervention is found effective.
**1. How can results from Phases 1 and 2 be used to develop an implementation strategy, process, or “playbook” by which interventions will be adopted and sustained in routine practice by existing providers?** a. Describe the extent to which the trial was pragmatic and its implications for sustainment (i.e., if it was not pragmatic, how will might this impact sustainment and scale-up of the intervention or treatment? b. Describe the training required among existing and new providers to deliver the treatment or intervention c. Describe the clinical processes required to maintain the treatment or intervention in routine practice and what additional administrative changes might be needed (e.g., addition of treatment into VA national formulary, lab tests, additional clinic time or procedures required to deliver treatment), where the intervention will take place (e.g., primary care, specialty, etc.), and how sustainable the intervention is (i.e., consider resources and staffing once the trial ends). d. Determine which measures should be used to monitor use of the intervention or treatment in routine practice (e.g., from existing EHR data, addition of variables to the formulary/EHR etc. e. Identify how sustainment of the intervention can be tracked over time (e.g., dashboards, surveys). f. Consider how qualitative data from leadership or interested parties can be used to understand the factors that may help or hinder future “real-world” spread, implementation, or sustainment and how there may be unique equity and diversity considerations during these different phases g. Describe if certain implementation strategies are more likely to sustain this intervention over time and have sustained effects over time. h. Consider how results will inform future interested parties’ acceptance of the intervention. i. Collect information on cost, including opportunity costs, and burden from different interested party perspectives, patient engagement, satisfaction, barriers, and health equity. *Note: Qualitative interviews or surveys with intervention-interested parties during phase 1 and phase 2 could be used to understand potential sustainment of the* *intervention* ^3^ *The implementation of the intervention was left to the individual VA Medical Center sites and there was no component in the proposal to address this. The Study* *Chair continued carrying the message of the effectiveness of this intervention through clinical care interested parties, additional research into IPS cost efficacy, other funded implementation projects, and various dissemination efforts.* *As shown in follow-up research study, IPS is sustainable and cost-effective with good social return on investment. Sustainability can be measured through existing* *data collection from VA Northeast Program Evaluation (NEPEC) as is done for IPS service delivery for the seriously mentally ill population and other PTSD programs. Qualitative data from key leadership and interested parties are important factors in real-world spread. In a resource-constrained healthcare system such as VHA, the main barriers to implementation of IPS include resource allocation (FTE) and need to replace outdated vocational rehabilitation services that have no evidence-based rationale; however, this strategy is threatening to the status quo within local VA facilities and at the national-level program office. The traditional vocational rehabilitation services place Veterans in low-wage transient jobs typically in the VA medical center for “work hardening experiences” prior to transitioning them without much follow-along support into the competitive job environment. VA facilities may have a secondary gain in having a labor pool for low-wage low-skill jobs, such as Veterans with disabilities, to meet the needs of the facility confronted with short staffing in areas such as housekeeping and grounds maintenance.* *These issues trigger political tensions within the national-level program office that are difficult for an expert investigator assigned to a local VA to navigate* *withouthigh-level interested party support.*
**Phases 1–3. Planning for Dissemination: How will intervention and implementation information and trial results be shared with others to increase adoption of the intervention?** Throughout all 3 phases of implementation planning, teams should consider the types of information that should be disseminated, to whom information should be disseminated, and how information should be tailored to address equity and diversity considerations of different individuals and healthcare settings. Dissemination is important for increasing awareness of the intervention, offering opportunities for bidirectional communication, and accelerating the buy-in/adoption/uptake of the intervention by providers, patients, and/or healthcare systems.
**1. What information should be disseminated during Phase 1 “Planning, Framing, and Aligning Interested parties” (e.g., increase intervention awareness and buy-in among interested parties)?** a. Identify to whom information should be disseminated by creating a visual display or mapping of interested parties b. Consider dissemination as an opportunity for bidirectional communication to and from interested parties to inform initial planning *This was done in the context of planning with the interested parties present at the CSP planning meetings. In hindsight, more could have been done at the* *national level to accelerate buy-in and uptake of the intervention.*
**2. What information should be disseminated during Phase 2 “Implementation Process and Data Collection” (e.g., Share implementation plans and support tools with key interested parties)?** a. Identify to whom information should be disseminated by creating a visual display or mapping of interested parties b. Consider dissemination as an opportunity for bidirectional communication to and from interested parties to inform implementation *Fidelity monitoring visits would happen semi-annually at each site. Feedback was given during/after those visits. In addition, feedback and data on this were* *presented to all site research personnel at the Study’s Annual Meetings. On a regular basis, participating VA Medical Centers would receive feedback from the IPS fidelity monitor as it related to the implementation of the intervention during the execution phase of the project. He would also share implementation support tools and encourage planning for post-study sustainment with facility leadership during de-briefing meetings following semi-annual IPS fidelity monitoring visits. The regional IPS mentors would use this information to provide technical assistance by weekly teleconference during bi-monthly on-site field mentoring visits. Once a year, all participating VA Medical Center personnel would be informed of such information through data reports presented at in-person Study Group meetings.*
**3. What information should be disseminated during Phase 3 “Planning for Sustainment for Effective Trials” (e.g., maintain priority and awareness of the intervention among key interested parties)?** a. Identify to whom information should be disseminated by creating a visual display or mapping of interested parties b. Consider dissemination as an opportunity for bidirectional communication to and from interested parties to inform sustainment *During sustainment, the actual outcomes of Veterans participating in IPS services should be shared (qualitative and quantitative outcomes) with interested parties* *so that awareness and support for the program can be maintained and new research findings can be considered to adapt the program so that it achieves the highest impact possible. Plans should include an executive roundtable including medical center directors/leadership, Veterans Affairs Central Office, Secretary’s office, and VA Center for Innovation.*
**4. Throughout all three phases, what is the plan for how and when intervention information and trial results will be disseminated, tailored, and communicated to interested parties and potential non-VA adopters? The following considerations may differ for each of the three phases:** a. Identify various “passive” publication opportunities (e.g., peer-reviewed journals, other publications) b. Identify more “active” strategies for disseminating information and results, which have been found to be more effective in reaching key interested parties (e.g., briefings to VA local, regional and national leaders-CMO national calls, VISN meetings, program office meetings, news or social media outlets, workshops, meetings) c. Determine if there are local or national opportunities to present trial results (e.g., professional conferences, VHA Cyber-seminars) d. Consider how intervention materials and results can be disseminated to diverse interested parties (e.g., clinical and non-clinical audiences, patient groups, Veteran and Family Advisory Councils). e. Develop other products that could be used to disseminate trial results (e.g., websites, toolkits) *Note: Consider developing an interested parties map to identify and engage relevant interested parties in future implementation processes. B-E will likely offer* *more opportunities for bidirectional communication* *The plan for dissemination was limited to publicity of the study results, to include peer-reviewed manuscripts, presentations at international conferences, talks by* *the Study leadership at various VA Medical Center facilities and outside healthcare systems, VHA Cyber-seminars, VA Center of Innovation meetings, and VA regional and local mental health summits.*
**5. What are the plans for using the intervention in future implementation research?** *Note: Consider funding from a VA QUERI center, VA Office of Research and Development/Health Services Research & Development (HSR&D) research study, VA* *national program office policy (use the Evidence Act), VA Diffusion of Excellence, National Institutes of Health-funded research, or foundation grants.* *Although this was not considered in the context of the CSP research project, the study Chair and IPS fidelity monitor went on to launch a 3-year pay-for-success* *social impact bond implementation of IPS at four VA Medical Centers in the Northeast area, called Veteran CARE. Dr Davis and members of the CSP Coordinating Center also conducted an HSR&D-funded cost efficacy study utilizing CSP data. Dr Davis and her work in IPS has been featured in VA Diffusion of Excellence marketplace. Dr Davis was also subsequently funded by VA Rehabilitation Research & Development to test the efficacy of IPS in a primary care setting for Veterans with a broad range of non-psychotic mental health conditions.*

1 While the tool was designed for use in both efficacy and effectiveness trials, effectiveness trials will have broader and more involved implementation methodology since they focus on answering the question: do the intervention benefits hold true in real-world clinical settings? In contrast, the tool will be more limited in efficacy trials since they focus on the answering the question: does the intervention work in a highly controlled standardized setting? Therefore, throughout the tool, we have indicated sections that may not be useful during efficacy trials.

2 See Frayne SM, Pomernacki A, Schnurr PP. *Women’s Enhanced Recruitment Process (WERP): Experience with Enhanced Recruitment of Women Veterans to a CSP Trial.* Invited national VA HSR&D CyberSeminar, presented November 15, 2018. https://www.hsrd.research.va.gov/for_researchers/cyber_seminars/archives/video_archive.cfm?SessionID=3565

3 Denotes overlap between Planning, Framing, and Aligning Interested Parties Phase and Implementation Process Data Collection phases.

## Materials and Methods

### Implementation Guidance and Planning Assessment Tool for Clinical Trialists

The IPA Tool was developed by authors CPK, LMK, and ALN and is informed based on principles outlined in the Implementation Roadmap developed by the ORD Quality Enhancement Research Initiative (QUERI) as well as main components and principles of the field of implementation science [2,15-17]. The IPA Tool was developed through a systematic process by an interdisciplinary team with expertise in implementation science, clinical trials, program evaluation, and qualitative methods; team meetings with an organized set of agendas over a period of time were used to develop and refine the tool.

**Fig. 1.**
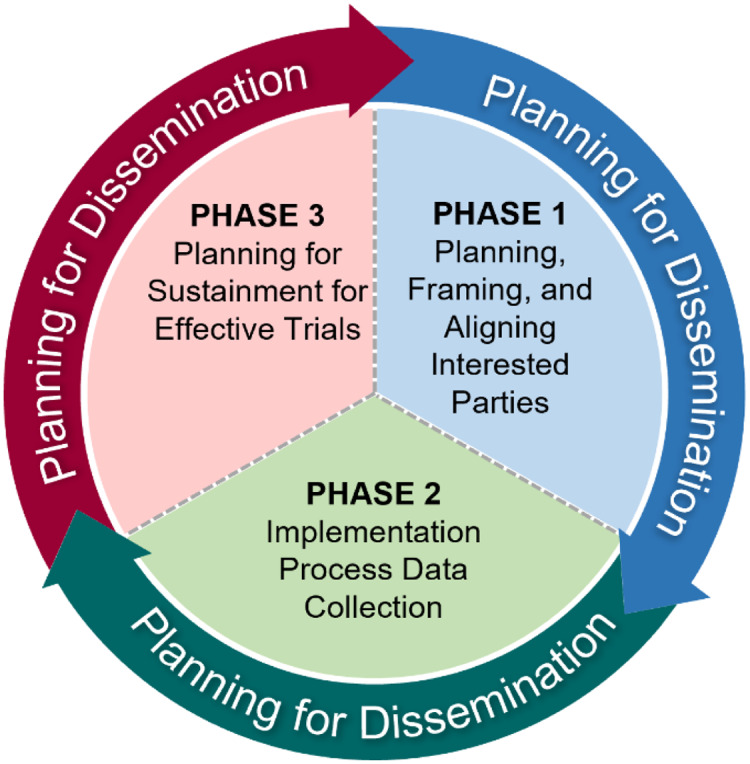
Framing for the Implementation Planning Assessment Tool.

The IPA Tool emphasizes three phases that are adapted from the QUERI Implementation Roadmap [18] of incorporating implementation science to accelerate the adoption of interventions into routine care (Fig. 1). The first phase, “Planning, Framing, and Aligning Interested Parties,” involves identification and garnering of input from multi-level (e.g., local, regional, and national level) interested parties who have a vested interest in the trial’s results and potentially the leverage to incorporate results or effective treatments into routine practice via organizational changes. Importantly, interested parties should include Veterans or patients to provide input on treatment use from an end-user perspective. In clinical trials, interested parties could include, but are not limited to, frontline staff, clinicians, nurses, leadership at different levels including national, regional, and local, clerks, check-in staff, Veterans or other patients, caregivers, operational leaders, and policymakers. See Table 3 for a broad spectrum of potential partners who may have a direct or indirect role in supporting the design, delivery, or receipt of the intervention. The second phase, “Implementation Process Data Collection,” involves planning and assessment by clinical and research leaders that will promote uptake of the intervention if found effective and the enactment of an IPA Tool. The third phase, “Planning for Sustainment for Effective Trials,” takes results from phases 1 and 2 to outline a process by which trial results and interventions (if proven effective) will be adopted in routine practice. Throughout all three phases, the assessment team should also be “Planning for Dissemination,” which involves sharing information about the intervention, implementation, and trial results to increase uptake among key interested parties.

**Table 3.** Interested parties

Getting the right people involved in an implementation initiative is a critical first step. Interested parties make up the broad spectrum of potential partners who may have a direct or indirect role in supporting the design, delivery, or receipt of the intervention.
In clinical trials, interested parties could include, but are not limited to: • Frontline staff, • Clinicians, • Nurses, • Leadership at different levels including national, regional and local, • Clerks, • Check-in staff, • Veterans or other patients, • Caregivers, • Operational leaders, • Policymakers, • Healthcare systems, • Professional societies, • Funders, • Patient advocates, • Community organizations, • Information and technology services, • Other hospital employees (housekeeping, medical assistants, engineering, etc.).

After the tool was developed, authors LD and TK applied the tool retrospectively, reflecting on a recently completed CSP clinical trial that was selected based on some characteristics for which the tool is intended to apply and highlighting concrete impacts of missed opportunities that could have been addressed had the tool been used throughout the life of the trial (Table 2).

## Results

### Implementation Planning Assessment Tool

This tool was designed to help trialists systematically think through essential components of an implementation science-informed process plan that is flexible enough to address the wide spectrum of research questions evaluated by clinical efficacy and effectiveness trials. While the tool was designed for use in both efficacy and effectiveness trials, effectiveness trials will have broader and more involved implementation methodology since they focus on answering the question: do the intervention benefits hold true in real-world clinical settings? In contrast, the tool will be more limited in efficacy trials since they focus on the answering the question: does the intervention work in a highly controlled standardized setting? Therefore, throughout the tool, we have indicated sections that may not be useful during efficacy trials. Trial proponents are encouraged to consider this tool as a prompt (i.e., at the time of study design/planning) to consider key issues to help promote consistency and rigor across the implementation plans developed for different trials. The tool will enable a way to capture real-world data to understand the intervention’s use and effectiveness over time as well as generate a comprehensive plan for ensuring trial results will be utilized by frontline providers in routine care settings upon study conclusion.

The intention is for this tool to be completed as an iterative process and the teams and individuals can and should refer to the tool at different points in time throughout the trial. For example, planning from phase 1 will impact later work in phase 2 when the team will speak with different key interested parties, possibly during team or advisory planning meetings. Then those areas of the table within phase 2 can be completed as the team moves forward with the trial.

#### Phase 1: Planning, framing, and aligning interested parties

In a non-clinical trial setting, the first phase would be referred to as “pre-implementation work.” However, within the clinical trial setting, we have labeled Phase 1 as “Planning, Framing, and Aligning Interested Parties” because using the term “implementation” would be a misnomer, since in the context of a clinical trial, effectiveness has not yet been determined and equipoise must be protected during the course of the trial. Phase 1 assesses the many complex factors that influence implementation or uptake of new programs, in addition to their success or failure. Formative evaluation (FE), defined as a rigorous assessment process designed to identify potential and actual influences on the progress and effectiveness of implementation efforts, is an essential means to systematically approach this complexity [19]. FE systematically examines key features of the local setting, detects and monitors unanticipated events, and adjusts, if necessary, in real-time, and optimizes implementation to improve potential for success [20]. This understanding is essential for efforts to sustain, scale up, and disseminate any new EBI. Otherwise, there is potential for failure to account for specific contextual issues in program implementation.

Phase 1 includes the first steps of FE in the IPA Tool beginning with asking what is the challenge or issue that the treatment or intervention is trying to solve, and what are the core elements that are hypothesized to achieve its desired effect on health? This needs to be explicitly mapped out and the interested parties involved in the intervention need to be identified. Staff time and resources should be protected to support Phase 1-3 work, for hereafter these staff will be referred to as “assessment planning staff.” While CSP has expertise in identifying national interested parties, such as national clinical program office leads, the Implementation Lead or other staff helping to complete the assessment will assist with identifying local interested parties including patient, clinical and non-clinical staff, and policymakers. Frontline users (clinical and non-clinical staff) should provide input into the deployment of the treatment or intervention. Veterans (or other patient populations as appropriate) should be asked to provide feedback on equity, barriers, and satisfaction. Organizational leaders who will decide about eventual program adoption should also be included in planning efforts to understand their concerns and priorities. Assessment planning staff will also help inform, as appropriate for the trial design, the design of the intervention to be implemented (design-for implementation, user-centered design) and deployment of the intervention. Contextual factors, such as competing demands, belief or lack of belief in evidence, loyalty to usual care modalities, available resources, leadership support level, clinical and/or operational policy, and frontline buy-in, will be assessed and documented. Barriers and facilitators to implementing the intervention will be assessed through mainly qualitative data including interviews, focus groups, conversations, and advisory call or meeting notes. Preliminary plans for the intervention’s sustainment (once the trial ends, if found effective) should begin. The plan should take into consideration any administrative or policy changes needed at the national and regional levels. These can include, but are not limited to, formularies, labs, electronic health record fields, national directives, or other services policies, budgeting, the time, tools, and training required by clinicians at the frontline to deliver the intervention, location for new service delivery (e.g., primary care, specialty care clinics, Community-Based Outpatient Clinics), and Veteran level of interest, time, and burden required to participate in the evidence-based intervention (e.g., visits, required lab tests, medications).

#### Phase 2: Implementation process data collection

Phase 2 involves the ascertainment of factors affecting the use of the CSP intervention at the routine practice level, notably through information on provider and patient acceptance, implementation and intervention costs and organizational factors, and fidelity to the implementation of the intervention or treatment, where relevant. Within clinical trials, the intervention is typically implemented within a controlled research context. Even when pragmatic trial design principles are included, there still remains some elements that do not perfectly mirror a clinical practice context. While a clinical trial may hire staff to deliver the treatment or intervention, the data collected for a clinical trial will likely be different than data collected from routine clinical providers. Both perspectives will be essential and data collection should incorporate both.

Critically important will be the continuation and protected time of the implementation assessment team, including involved staff. Implementation plans initiated during Phase 1 will now be finalized. The key barriers and facilitators identified during Phase 1 will be used to select and define implementation strategies. The implementation strategy dose [21] along with the implementation outcome most likely to be affected by each strategy should be documented. Likewise, justification should be provided for the choice of implementation strategies, including theoretical, empirical, or pragmatic justifications. Adaptations or resources will be deployed that are necessary to fit for local contexts. This will include planning proactively how the intervention may need to be adapted going forward to better fit real-world contexts (i.e., What adaptations are needed to be able to implement the intervention?), the identification of strategies to support the people and clinical interested parties delivering the intervention to Veterans [22] (i.e., Which implementation strategies will help overcome barriers and improve implementation of the intervention?), and determination and planning for evaluation of the benchmarks of successful implementation should take place.

Phase 2 will use a combination of both qualitative and quantitative data collection, known as mixed methods data collection [23], to *formatively* identify and evaluate how multi-level contextual factors at the Veteran-, patient-, clinician-, facility-, or health system-level serve as barriers or facilitators to quality delivery and receipt of the trial intervention over time. Secondarily, some formative process data can be shared in trial staff meetings to make iterative improvements to key protocol steps as a *quality assurance* step to address unanticipated research challenges, such as achieving participant recruitment and enrollment goals or fostering adherence to clinical lab monitoring.

#### Phase 3: Planning for sustainment for effective trials

Upon trial completion, the IPA team can use data collected by mixed methods approaches to help make a *summative* judgment regarding the influence of context on study outcomes. This is crucial to determine how the trial results will be used in routine care once the study has ended, while also developing a plan to implement interventions if proven effective. Key lessons from this formative and summative process evaluation can be used in the future to help clinicians and healthcare sites deliver the intervention protocol more effectively, thereby accelerating the uptake of interventions found to be effective following validation of clinical impact.

If the intervention is found to be effective, it is important for there to be sustainment planning activities in place so that continuity of patient care through the intervention is maintained. Sustainment planning will help evaluators and researchers understand how the intervention will be used in routine care once the study has ended. Accordingly, results from Phase 1 and 2 will inform clinicians and healthcare sites in understanding how to deliver the intervention protocol more effectively, make appropriate adaptations, and sustain the intervention over time. The implementation team should identify how sustainment and further dissemination (e.g., scale-up and spread in sites beyond the original study sites) can be tracked over time through tools such as surveys or dashboards. If Phase 1 and 2 data have shown that certain implementation strategies will be more effective at sustaining the intervention over time, then those strategies should be utilized at this point.

### Phases 1–3: Planning for Dissemination

The implementation planning team should consider how various types of information can be disseminated and to whom information should be disseminated throughout the three phases described in the IPA Tool. During Phase 1, dissemination focuses on initial sharing of information about the intervention to increase awareness and buy-in from key interested parties. During Phase 2, dissemination efforts are focused on sharing implementation plans and tools with key interested parties. During Phase 3, dissemination includes, but is not limited to, sharing information with key interested parties that will support sustainment, and possibly spread, of the intervention. In addition, after the clinical trial ends, the IPA team will facilitate dissemination and translation of trial results beyond traditional strategies of journal publication and study summaries to increase adoption of the intervention, thereby improving the clinical care of Veterans and/or by improving VHA policy. Dissemination helps increase opportunities for bidirectional communication between the implementation assessment team and interested parties and the likelihood that results from this research will be adopted by national program offices and national organizations via evidence-based guidelines.

### CSP Case Study Using the IPA Tool

The Veterans Individual Placement and Support Toward Advancing Recovery (VIP-STAR) was a VA CSP multicenter, prospective, randomized clinical trial comparing the effectiveness of Individual Placement and Support (IPS) vs. usual care transitional work in unemployed Veterans with a diagnosis of PTSD. The study found that, as hypothesized, more Veterans in the IPS group became steady workers (primary outcome) and earned more income from competitive jobs (secondary outcome) over the 18-month follow-up compared with the transitional work group [12].

From its inception, the study incorporated a collaborative partnership between the clinical and research team(s) at each of the participating sites. Sites were selected based on qualifications of local site investigators, a previous track record of success with implementation of IPS for the serious mentally ill population, and a well-established and operational transitional work program. While the CSP Planning Committee did not include an implementation scientist, members did include IPS trainers, fidelity monitors, program evaluators, PTSD clinicians, and vocational rehabilitation experts. The IPA Tool would have provided the clinical trial planning committee with a roadmap to formulate a comprehensive inventory of employment-related resources and implementation challenges within the VA as well as approaches toward strategic involvement of VHA policy and clinical program leaders to align with the conclusions and results of the study. Using the tool may have better ensured that the positive results from the trial would more efficiently transform future service delivery. As indicated in the IPA Tool, interested parties should be provided with an opportunity to give input into the trial design and the structure of the treatment conditions so that end results could be trusted and embraced by all rather than elicit a threat to the status quo at the conclusion of the study (see Phase 1, item 6; Phase 2, item 3; Phase 3, item 1 in Table 2). In addition to input, the tool would have provided engagement and ownership of a robust process by both the VA research enterprise and VA clinical services acting in synergy so as to facilitate commitment to ensuring deployment of interventions proven to be effective/efficacious within a short period of time post-trial conclusion. Involvement of the end-user of services, that is, the Veteran with PTSD-related employment issues, and medical center directors represent other missed opportunities in pre-trial planning that could have been addressed using the IPA Tool. As noted in the case example, a structured study-wide process to help attain broad implementation (e.g., implementation at all study sites once the clinical trial ends) was not designed prior to study launch for this intervention. This deficit in planning for implementation likely negatively impacted the sustainment of IPS at the 12 local sites. A key barrier to post-study implementation was lack of funding for the local site IPS specialists designated to serve the PTSD patient population. Precedent existed for VA to support an enterprise-wide rollout of IPS for Veterans with psychotic disorders (2005). The investigators assumed that a similar funding stream would materialize after the evidence emerged for the PTSD population. Had the tool been used prospectively, cost planning would have occurred that likely would have enabled planning to circumvent this barrier. Leadership changes at the national level contributed to an attrition in resources for IPS sustainment which resulted in diminished focus on expanding services and/or ongoing quality monitoring across the board. No benchmarks of successful implementation were drafted and the trialists note (see Table 2, phase 2, section 5) that future implementation projects should include these measures of success: number of Veterans referred for and engaged in IPS services per month, number and duration of competitive jobs gained per participant, income earned per participant, patient satisfaction surveys, IPS fidelity scale scores, and job satisfaction scores.

## Discussion

VA considers itself a Learning Health System dedicated to applying evidence to improve healthcare [14,24,25]. Implementation science is an important component of VA’s transition to a LHS because it involves strategies to improve the rapid uptake of effective treatments into real-world practice. Incorporating principles of implementation science in clinical trials through the IPA Tool could help achieve Learning Health System goals [26] and ensure that research findings are relevant to health systems and patient and community interested parties. This assessment, which notably includes recurring ascertainment of patient preferences and perspectives, will help uncover important variables that impact quality of life for Veterans and patients and willingness to use an intervention, particularly for those with health behavior components or requiring patient consent. Likewise, the evaluation of clinician and non-clinical staff perspectives can help with scale-up and spread of successful trials by uncovering and weaving around clinician barriers. Through use of the IPA Tool, perspectives of the interested parties, including engagement of hospital leadership on whom uptake and implementation depends, will be understood, which will help to identify ways to navigate around competing priorities and constrained resources in health settings. Although this tool was designed and applied retrospectively in a VA healthcare setting, the principles on which the tool is based are drawn from the field of implementation science as a whole and, therefore, broader than the VA context alone. The areas of focus indicated in the tool can be applied to other healthcare settings with little work to adapt. Even in the case of a negative or ambiguous trial result, lessons can be learned about barriers and facilitators that can be extrapolated to like organizational contexts or similar trials. An implementation plan is designed and integrated into a study protocol a priori and agnostic of subsequent study results. Trial results that are either inconclusive or fail to reach statistical significance should not discount the utility of an implementation plan. An intervention could fail to show efficacy/effectiveness either because it simply does not work as assumed (effect size overestimated; mechanism of action incorrectly considered) or it could not work (uptake and adherence miscalculated). Having an implementation plan integral to the protocol sheds light on the latter issue. If barriers to uptake and adherence to the intervention are adequately addressed in the context of an implementation plan and yet the results do not provide evidence of its efficacy/effectiveness, this could point to the direction of an ineffective intervention. At the same time, implementation helps identify, upfront, structural, and process barriers that could potentially inform testing anew of a new/different/more efficacious/effective intervention.

Most current academic promotion pathways do not incentivize implementing treatments or findings into practice [14], nor do common funding mechanisms include support for implementation activities. The types of activities described in this manuscript and throughout the IPA Tool necessitate significant time and follow contrary to the general manuscript or grant funding mechanism. Resource allocation to programs such as that of QUERI program [8] and the Implementation Research Project (IRP) mechanisms needs to grow to incentivize these types of scientific inquiry. Likewise, funding agencies such as VA, NIH, and AHRQ should consider requiring plans for the phases covered in this IPA Tool for sustainment, scale-up, and spread, if the trials are found to be effective. This requirement would also necessitate that funding agencies increase funding to support development of implementation plans.

The CSP breadth of inclusion of implementation plans is evolving and spreading throughout their trials and over time. As the clinical trials arm operating within the VA’s large learning healthcare system, CSP is in a unique position to bridge, in a bidirectional way, the clinical and research arms of such a system. Utilizing implementation science and its robust methodology and tools, CSP can support the design, operation, management, execution, and analysis of definitive clinical trials, while at the same time ensure implementation, sustainability, and continuity of effective interventions resulting from its clinical trials. In addition, the ability of the clinical side of the VA system to identify clinically significant questions and then generate testable hypotheses with support from and in true partnership with the built-in CSP clinical trials operation allows for impactful research to be strategically planned and executed toward fulfilling the mission of improving Veteran healthcare through quick deployment and clinical setting adoption of such evidence-based research.

Given the case example represents a team that retrospectively applied the tool, we cannot yet provide an accurate estimate of how much effort will be needed to complete it prospectively, as intended. Our team is working with 2 clinical trials that are currently applying the tool prospectively and will have more information in the future about the time and effort needed to complete the tool. As mentioned earlier, the intention of the tool is not that the team should complete it all at once, but rather revisit as needed. It is up to the individual/s and team to determine how they want to use the tool and to what extent. Application of the tool will take some time. However, these efforts should help teams better integrate clinical trials and original research into clinical practice by proactively facilitating adoption of research results into routine care in a sustainable way.

### Limitations

There are several limitations to this manuscript. First, the IPA Tool was designed to provide trialists and interested parties in the outcomes with practical guidance on how to include implementation planning in clinical trials. However, the IPA Tool may not describe all aspects of implementation planning. Therefore, we encourage users of the IPA Tool to consider how to tailor the steps in the checklist dependent on the type of trial, effectiveness, or efficacy, as the applications can vary depending on trial type. Second, the retrospective use of this tool for the case example had limitations since some of the phases were not addressed during the CSP trial. However, the CSP trialists who completed the case example were able to reflect on the phases that were completed and then describe missed or future opportunities for improving implementation planning. The team believes that if the IPA Tool has been available much of the trial-and-error approach could have been avoided. In addition, since CSP trials have only recently been matched with implementation scientists, they did not have data to complete all three phases of the IPA Tool. Though the IPA Tool may have a greater impact if used prospectively, our case example suggests there are also benefits of using the tool retrospectively.

## Conclusion

The IPA Tool brings a ready-made list of necessary steps for clinical trialists aiming to improve implementation, including scale-up and spread, of effective, clinical trial-tested interventions in healthcare settings. The IPA Tool was designed to help catapult the field of clinical trial inquiry into a new realm of applied practice. Likewise, the IPA Tool can also be utilized by practitioners, clinicians, and researchers who are new to the field of implementation science and the involved processes to help with grant writing and throughout the life of their implementation trials.
